# Clinical Characteristics, Cytogenetic Risks, and Prognoses of Young Multiple Myeloma Patients in the Era of Novel Therapies

**DOI:** 10.3390/cancers16234090

**Published:** 2024-12-06

**Authors:** Nathalie Borst, Gabriele Ihorst, Sina Wenger, Jan Räder, Ralph Wäsch, Monika Engelhardt, Michael Rassner

**Affiliations:** 1 The Department of Hematology, Oncology, and Stem-Cell Transplantation, Faculty of Medicine and Medical Center, University of Freiburg, 79106 Freiburg im Breisgau, Germany; nathalie.gutjahr@uniklinik-freiburg.de (N.B.); sina.wenger@uniklinik-freiburg.de (S.W.); jan.raeder@uniklinik-freiburg.de (J.R.); ralph.waesch@uniklinik-freiburg.de (R.W.); monika.engelhardt@uniklinik-freiburg.de (M.E.); 2 Clinical Trials Unit, Medical Center-University of Freiburg, Faculty of Medicine, University of Freiburg, 79085 Freiburg im Breisgau, Germany; gabriele.ihorst@uniklinik-freiburg.de

**Keywords:** multiple myeloma (MM), younger age, novel agents, stem cell transplantation, risk factor assessment

## Abstract

**Background/Objectives:** In recent years, there have been significant advances in the understanding and treatment of multiple myeloma (MM). Despite this progress, there is still limited information on the disease in patients aged 50 or younger, including the impact of young age on disease characteristics, treatment, and outcome. **Methods:** In this retrospective study, we analyzed 68 newly diagnosed MM patients aged ≤ 50 years (y) who had undergone at least one peripheral blood stem cell transplantation (PBSCT). Additionally, we reviewed data published during 2008–2022 and compared these to our cohort. **Results:** Of note, the disease characteristics in our cohort were similar to those in older patients. However, the incidence of bone lesions was higher in younger patients (84%). Moreover, 33% had LC-only MM and 7% had high-risk (del17p, t(14;16), t(4;14)) cytogenetics. Advanced ISS and R-ISS II/III were observed in 57% and 78%, respectively. Therapy was intense, with 53% of patients undergoing ≥2 SCTs. Median follow-up was 75 months, median progression-free survival was 57 months, and median overall survival (OS) was not reached. The 10-year OS rate was 72%, with only 19% succumbing to the disease. Notably, no specific therapeutic regimen or risk factors for worse outcomes were identified through uni- or bivariate analyses, even in subgroup analyses of younger patients aged ≤ 40 y. **Conclusions:** Our, and prior, results of young (<50 y) and very young (<40 y) MM patients underscore the need for further comprehensive studies focused on this significantly affected cohort.

## Simple Summary

Therapies for patients with multiple myeloma (MM) have substantially improved within recent years. There are some MM patients that are very young and fit (≤50 years), yet heavily affected by this life-changing condition. For these patients, the outcomes, i.e., progression-free survival and overall survival, remain unsatisfactory with a rather unchanged impact on age-adjusted morbidity. A look into the literature for “MM” and “young age” resulted in ambiguous conclusions. Therefore, we decided to analyze the clinical parameters of younger MM patients to possibly identify additional risk factors indicating worse outcomes. Additionally, we compared our study with the published literature of the last decade.

## 1. Introduction

Multiple myeloma (MM) is a clonal plasma cell malignancy, comprising approximately 10% of all hematological diseases. While the median age of onset is around 70 years [1], MM is notably rare in younger individuals affecting only 2% of patients ≤ 40 years [2]. Detailed data on the disease characteristics, prognostic factors, and therapy implications for this younger demographic are limited. The disease impacts young patients at critical stages of their lives, often complicating treatment due to family and professional obligations. Additionally, responses to treatment and patient outcomes in this subgroup have been inconsistently reported [2,3,4,5].

The aim of this study was to thoroughly examine this population at our Comprehensive Cancer Center Freiburg (CCCF) and to identify additional prognostic factors for progression-free survival (PFS) and overall survival (OS). Additionally, we conducted a comprehensive review of the literature and compared our findings with previous data [2,3,4,5,6,7,8,9,10].

## 2. Materials and Methods

In a retrospective study, we analyzed 68 consecutive MM patients ≤ 50 years of age at first presentation at our CCCF between 1 January 2010 and 31 December 2020. All patients had undergone at least one peripheral blood stem cell transplantation (PBSCT), the standard treatment option for young and fit MM patients at our center and globally [11,12]. Patient data were extracted from the CCCF/University Hospital of Freiburg (UKF)-transplantation database as described previously [13]. Patient characteristics, therapies, responses to treatment (according to IMWG), and outcomes (PFS and OS from the first PBSCT) were assessed descriptively. Survival probabilities were estimated with the Kaplan–Meier method. Univariate Cox regression models were applied to investigate the impact of individual patient characteristics on PFS and OS. The two most relevant parameters were inspected in a bivariate Cox model. A *p*-value < 0.05 was considered statistically significant. This study was carried out according to the Guidelines of the Declaration of Helsinki and Good Clinical Practice. All patients gave written informed consent, and analyses were performed in accordance with the institutional review board guidelines. The trial protocol was approved by the ethics committee of the UKF (EV 81/10, 27/14, 20/15).

## 3. Results

### 3.1. Patient Characteristics and Risks

The disease characteristics of 68 patients are presented in Table 1. The median age was 47 years, with 11 patients aged 40 years or younger, and the latter with a median age of 39 years. Male vs. female distributions were observed at 61% and 39%, respectively. The myeloma paraprotein subtypes were predominantly IgG (48%) and light chain (LC)-only (33%), with 65% of the LCs being kappa LCs. Advanced ISS and R-ISS II/III were present in 57% and 78% of patients, respectively. In line with these findings, the median bone marrow infiltration was substantial at 50%. High-risk cytogenetics (HRCG), as per the CCCF/UKF definition (t(4;14), t(14;16), t(14;20), del17p, hypoploidy, c-myc, or chromosome 1 aberration) [13] and as defined by the IMWG (del17p, t(14;16), t(4;14)), was found in 44% and 7% of patients, respectively.

Laboratory findings at initial diagnosis were similar to those observed in the general, typically older MM population (Supplementary Table S1A). The peripheral blood reconstitution of lymphocyte subsets after PBSCT was also comparable (Supplementary Table S1B).

Regarding the CRAB criteria, 84% of patients had detectable osteolysis at ID. Additionally, anemia, renal impairment, and hypercalcemia were substantial with 38%, 28%, and 19%, respectively. The majority of patients had one or two CRAB symptoms, while 12% had three symptoms and another 12% had all four. The prevalence of three or four CRAB criteria was significantly more substantial than previously reported by our group [14]. The median Karnofsky Performance Status (KPS) was 80%, while the median Revised Myeloma Comorbidity Index (R-MCI) was 4, both aligning with prior data in MM (the latter indicating an intermediate-fit status) [15]. Specifically, 44% of patients were classified as fit, and 56% as intermediate fit. Owing to the younger age and fewer comorbidities, no patient was classified as frail.

Treatment details are also summarized in Table 1. Induction therapy comprising proteasome inhibitor (PI)-containing triplets, with bortezomib, cyclophosphamide, and dexamethasone (VCD), was administered in 76% of patients, according to DSMM XI study protocol. This was a standard German induction regimen, prior to the adoption of the daratumumab–bortezomib–lenalidomide–dexamethasone (D-VRd) regime from the PERSEUS study [11], which is now the new German-wide and globally used standard induction regimen. At least two PBSCTs were performed in 53% of patients, with 31% of these patients receiving at least one autologous plus one allogeneic PBSCT (allo-SCT). Maintenance therapy, primarily with lenalidomide, was administered to 56% of patients.

**Figure 1:**
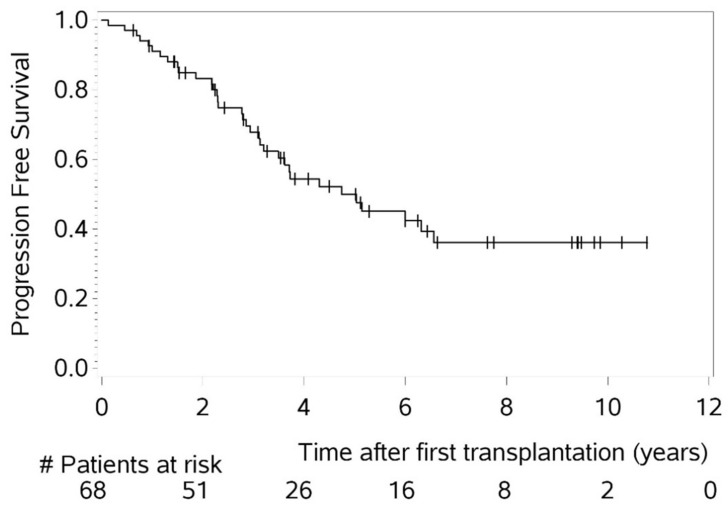
Progression-free survival (PFS) of entire cohort of patients ≤ 50 years of age (n = 68).

**Figure 2:**
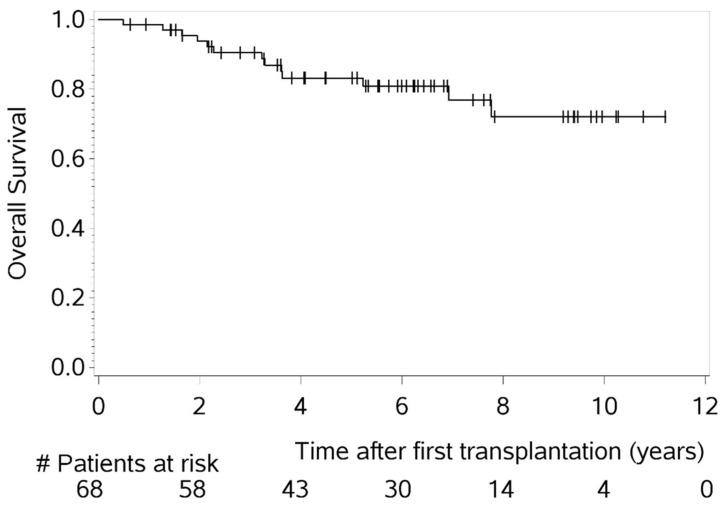
Overall survival (OS) of entire cohort of patients ≤ 50 years of age (n = 68).

A partial remission (PR) or better was achieved by 85% of patients after induction therapy. During the entire treatment course, every patient reached a response of at least PR at some point. Notably, 47% experienced progressive disease (PD) at least once and 12 individuals (18%) succumbed to MM. The median follow-up was 75 months (range 5–134), the median PFS was 57 months, and the median OS was not reached. The 5- and 10-year OS rates were 83% and 72%, respectively (Figure 1 and Figure 2).

### 3.2. Risk Factor Assessment

Univariate Cox regression analyses of potential risk factors for PFS and OS are depicted in Supplementary Table S2A, none of which reached statistical significance. A hazard risk (HR) > 1 for PFS was observed for LC-only, ISS stage II/III, HRCG, anemia, osteolysis, hypercalcemia, KPS ≤ 70%, and intermediate-fit (R-MCI 4–6) patients. Similarly, increased HRs > 1 for OS were identified for ISS II, osteolysis, and KPS ≤ 70% (Supplementary Table S2A). Via bivariate analyses, LC-only MM and HRCG appeared relevant with HRs > 1 for PFS. Supplementary Table S2B,C display 5- and 10-year PFS and OS estimates for various risk factors, none of which show statistical significance.

In order to determine whether the 12 patients (18%) who died from PD during our follow-up displayed specific risks that are especially pertinent in younger MM patients, we assessed their characteristics as outlined in Supplementary Table S3. We found that age, gender, MM type, ISS, CGs, therapy, and remission status after ASCT, tandem ASCT, or tandem ASCT/allo-SCT were similar to those of the entire cohort. However, disease progression (PD) occurred in all of these patients, with a median PFS and OS of 39 and 76 months, respectively. Thus, we did not identify specific risks that may predict death in these patients compared to the entire cohort.

### 3.3. Review of the Literature

We identified 11 publications covering MM patients ≤ 65 years, published between 2008 and 2022 (Table 2). Relevant patient and disease characteristics, therapy lines, responses to treatment, and outcomes of these publications as compared to our data are summarized therein. Notably, the cut-off age defining “young patients” was primarily 40 or 50 years (8/11 studies), with the number of included patients varying considerably (range 16–1689). There was a slight male predominance across these studies. Expectedly, the IgG subtype was predominant, and two studies confirmed our observation of an elevated proportion of LC-only MM in >20% [2,5]. The majority of patients had ISS stages I or II, with only our study assessing the Revised ISS (R-ISS). A high proportion of younger MM patients had bone lesions (≥75%), consistent with our findings (84%). Only one study provided the performance status (KPS) [5], showing a greater KPS (≥90%) similar to our findings, although Dhakal et al. reported a higher proportion of patients with KPS ≤ 70% (25% vs. 3% in our study) [5]. In line with our median KPS of 80%, the median R-MCI of “4” indicated that young patients were either fit or intermediate-fit, making them indeed very suitable for intensive treatment, including stem cell transplants (SCTs), tandem ASCT, or even tandem ASCT/allo-SCT [16].

Induction therapy in most studies consisted of triplets such as VCD, or bortezomib–lenalidomide–dexamethasone (VRd)/lenalidomide–adriamycin–dexamethasone (RAD), consistent with the DSMM XI and XIV studies [17] and in line with other centers now using D-VRd according to the PERSEUS study [11].

In some studies, patients received allo-SCT either upfront [2,4] or as a second-line treatment [2]. Most studies reported a response to treatment of at least PR, while two studies noted that patients predominantly achieved ≤PR [3,8]. Half of the studies described a prolonged OS for younger patients (median OS: 80 months to not reached) compared to older patients (median OS: 50 to 101 months) [2,3,4,6,7], whilst the remaining studies did not observe a difference in OS between younger and older patients [5,8,9,10,18].

**Table 2:** Review of the literature (2020–2023): comparison of disease characteristics, therapies, and outcome of prior “younger” MM cohort.

	**Borst et al., 2024 [This Work]**	**Kaloyannidis et al., 2022 [10]**	**Caulier et al., 2021 [2]**	**Bove et al., 2021 [3]**	**Duek et al., 2021 [19]**	**Pál et al., 2020 [18]**
Patients (n)/age cutoff (yrs.)	68/≤50	58/55	214/≤40	150/≤65	23/<50	16/40
Years of inclusion period	2010–2020	2010–2021	2000–2015	2011–2018	2009–2014	January 2006–December 2015
Median age (range)	47 (29–50)	46.5	37 (18–40)	57 (32–65)	41.5 (27–49)	39 (31–40)
Gender m:f (%)	61:39	52:48	64:36	52:48	74:26	63:37
IgG/IgA/IgM/LC only/asec. (%)	48/17/0/33/2	73/9/0/18/1	80/17/0.6/24/0	54/25/0/20/1	48/9/0/43/0	50/19/0/19/12
κ/λ/asecretory (%)	65/33/2	70/30/1	65/33/2	n.a.	61/39/0	n.a./n.a.
ISS I/II/III (%)	43/31/26	59 (I + II)/41/0/17	52/28/20	25/27/48	36/43/21 (n = 14)	44/31/25
R-ISS I/II/III (%)	22/60/18	n.a.	n.a.	n.a.	n.a.	n.a.
Durie and Salmon I/II/III/A:B (%)	10/15/75/78:22	n.a.	n.a.	3/11/85/n.a.	n.a.	n.a.
IMWG HR cytogenetics * (%)	7	12	18	18	0	31
C/R/A/B (%) **	19/28/38/84	n.a.	13/17/35/75	14/28/60/79	6/17/33/89	19/13/13/88
KPS > 90/80/≤70 (%)	41/34/25	n.a.	n.a.	n.a.	n.a.	n.a.
R-MCI 0–3/4–6/7–9 (%)	44/56/0	n.a.	n.a.	n.a.	n.a.	n.a.
Induction therapy (%)	VCD 76 RAD 12 VRD 8 Others 4	VRD/VTD/RD 53 VCD/VD 47 Others 0	VD/VCD/PAD 30 VTD/VRD 37 VAD/DCEP 26 Others 7	VCD 44 CTD 33 TD/VTD 11/5 Others 7	VCD/VD 43.5 VTD/VTD-PACE 26.1 VAD-TD 4.4 RD 8.7 NA 17.4	VTD/VTD-PACE 68 VAD 13 PAD 6 Thal/Dex 13
ASCT/2x ASCT/alloSCT (%)	47/22/31	91/0/0	77/23/25	55/0/0	100/0/0	88/0/0
Maintenance (%)	56	n.a.	75	23	7	63
Response to therapy:	40/50/10	37/24/39	38/34/28	19/22/59	63 (CR + vgPR)	13/50/37
CR/vgPR/≤PR (%)						
Median PFS (months)	36	49	41	40	12	n.a.
Median OS (months)	n.r.	n.r.	175	65; 80 with ASCT	n.a.	n.a.
5 years—OS rate (%)	83	75 (4-year OS rate)	84	n.a.	n.a.	83
10 years—OS rate (%)	72	53 (8-year OS rate)	59	n.a.	n.a.	n.a.
Median follow-up (months)	75	48	76	30	n.a.	n.a.
Conclusions	Disease characteristics comparable to typical MM elderly cohort; good outcome of younger patients ≤ 50 years with prolonged survival; risk factors for unfavorable outcome were not identified	Negative prognostic factors: female, high-level LDH, EMD at ED, ISS III	Disease characteristics comparable to elderly patients; multivariate negative prognostic factors for OS: bone lesions, high ISS, and HR-CG	Pts. ≤ 65 years have more aggressive disease and more advanced DS stage, extramedullary disease, osteolysis; response comparable to elderly pts (>65 years); OS better for pts < 65 years and prolonged by ASCT; risk factors for shorter OS: creatinine > 2 mg/dL, extramedullary disease, no ASCT, ≤vgPR after ASCT	Trend to poorer PFS for pat. with t(11;14)	No statistical results in comparison to subgroup >40; younger pts. underwent more maintenance
	**Jurczyszyn et al., 2019** [4]	**Dhakal et al., 2017** [5]	**Shin et al., 2017** [9]	**Jurczyszyn et al., 2016** [6]	**Cheema et al., 2009** [8]	**Ludwig et al., 2008** [7]
Patients (n)/age cutoff (yrs)	52/30	86/≤50	32/40	173/≤40	38/40	1689/≤49
Years of inclusion period	1989–2016	2000–2015	01/2000–02/2015	2000–2015	01/1990–08/2007	1981–2002
Median age (range)	28 (8–30)	46 (32–50)	37 (17–40)	37 (21–40)	37 (29–40)	36 (20–49)
Gender m:f (%)	67:33	81:19	59:41	40:60	61:39	61:39
IgG/IgA/IgM/LC only/asec. (%)	55/18/0/22/2	40/10/0/30/0	47/17/0/30	69/17/0/14/0	53/18/0/21/0	60/21/n.a./13/n.a.
κ/λ/asecretory (%)	n.a./n.a.	n.a.	n.a./n.a.	69/31/0	74/26	n.a.
ISS I/II/III (%)	68/15/17 (n = 47)	17/26/23	32/48/19	47/33/20	48/n.a./n.a.	39/35/27
R-ISS I/II/III (%)	n.a.	n.a.	n.a.	n.a.	n.a.	n.a.
Durie and Salmon I/II/III/A:B (%)	20/40/40/50:50 (n = 5)	n.a.	16/26/58/87:13		n.a.	8/232/60/85:15
IMWG HR cytogenetics * (%)	10	14	21	32	n.a.	Only del13 found/60 pts.
C/R/A/B (%) **	14/18/30/82	n.a.	28/13/29/87	16/25/31/82	23/25/67/76	33/15/37/48
KPS > 90/80/≤70 (%)	n.a.	43/52/3	n.a.	n.a.	n.a.	n.a.
R-MCI 0–3/4–6/7–9 (%)	n.a.	n.a.	n.a.	n.a.	n.a.	n.a.
Induction therapy (%)	PI-based 41 IMID-based 24 PI + IMIDs 21 Others 15	VR-based 23 V-based 44 T 7 R-based 10/9 Others 13	VAD/VTD/TD 67 VCD/VD 10 CTD/CD 10 MPT/MP 10 D 3	n.a.	VAD 66 D 13 MP 13 VAD + MP 2	n.a.
ASCT/2x ASCT/alloSCT (%)	62/0/3 (n = 34)	100/0/0	79/9/0	11/0/0	87/13/0	41/0/0
Maintenance (%)	n.a.	47	n.a.	n.a.	49	n.a.
Response to therapy:	38/12/50 (n = 34)	50/10/25/15 missing	64 (CR + vgPR)/23	33/23/45	29/0/71	n.a.
CR/vgPR/≤PR (%)						
Median PFS (months)	n.a.	n.a.	16	n.a.	22	n.a.
Median OS (months)	166	n.a.	61	n.a.	81.4	90
5 years—OS rate (%)	77	After 3 years 66	54	83	60	n.a.
10 years—OS rate (%)	n.a.	n.a.	n.a.	n.a.	43	43
Median follow-up (months)	86	33	64	n.a.	53	48
Conclusions	No univariate statistical results for OS; higher LC MM	Similar PFS and OS for younger (<50 yrs.) and older (>70 yrs.) pts; risk factors for OS: HR-CG; risk factors for PFS: HR-CG; no response to induction; age does not have an impact on MM outcome	No impact of age on OS; no difference between subgroups +/−ASCT; ISS I, lambda subtype, whole Ig trend result in prolonged OS	Disease characteristics of younger pts. (≤40 yrs.) comparable to pts. 40–60 yrs. but higher incidence of osteolytic lesions and HR-CG; risk factors for impaired OS: ISS III, response < CR; OS prolonged in younger pts.	No difference in OS and PFS compared to elderly cohort; CR or PR after ASCT = prolonged PFS but not OS; age has no impact on prognosis	Younger pts. have less unfavorable prognostic factors (CRAB) and more often ISS I = prolonged survival

Abbreviations and definitions: pts. = patients; n = number; m = male; f = female; Ig = immunoglobuline; LCs = light chains; asec. = asecretory; n.a. = not available; ISS = International Staging System; R-ISS = Revised International Staging System; IMWG = International Myeloma Working Group; CRAB = calcium, renal insufficiency, anemia, bone lesions; KPS = Karnofsky Performance Status; R-MCI = Revised Myeloma Comorbidity Index; VCD = bortezomib, cyclophosphamide, dexamethasone; RAD = lenalidomide, adriamycin, dexamethasone; VRD = bortezomib, lenalidomide, dexamethasone; VD = bortezomib, dexamethasone; PAD = doxorubicin, bortezomib, adriamycin, dexamethasone; VTD = bortezomib, thalidomide, dexamethasone; VAD = bortezomib, adriamycin, dexamethasone; DCEP = dexamethasone, cyclophosphamide, etoposide, cisplatin; CTD = cyclophosphamide, thalidomide, dexamethasone; TD = thalidomide, dexamethasone; VR = bortezomib, lenalidomide; V = bortezomib; T = thalidomide; R = lenalidomide; ASCT = autologous stem cell transplantation; allo-SCT = allogeneic stem cell transplantation; CR = complete remission; vgPR = very good partial remission; PR = partial remission; OS = overall survival; HR = high risk; CG = cytogenetics; DS = Durie and Salmon; PFS = progression-free survival. * Unfavorable IMWG: del17p, t(14;16), t(4;14); ** more than 1 CRAB criteria possible, therefore in sum > 100%.

## 4. Discussion

MM patients under the age of 50 years reveal a significant gap in basic and clinical research. So far, there have been no data that have found that the biology of MM in younger patients differs significantly from that in older patients. While numerous studies have explored the impact of age on cancer development, particularly in relation to immunosenescence [20], there is limited research specifically addressing age-related differences in the biology of MM. It is possible that in younger, generally more immunocompetent hosts, malignant clones may undergo more intense immunoediting [21], ultimately contributing to the development of MM. Such mechanisms could potentially explain less favorable outcomes in younger patients, though, to date, no studies have specifically investigated this phenomenon in MM. From a clinical perspective, there are only limited and inconsistent reports on younger MM patients in the existent literature [2,3,4,5,6,7,8,9,10,18,19]. This age group remains rare in the literature, and the observations presented in these studies often diverge, underscoring the need for a comprehensive investigation. Few studies have identified risk factors for unfavorable OS in young MM patients, such as advanced ISS stage, HRCG, bone lesions, hypercalcemia, absence of PBSCT, and achieving less than CR after the first PBSCT. Similarly, risk factors for PFS included HRCG and lack of response to induction therapy. However, these risk factors are also associated with poor prognosis in elderly MM patients [2,3,5,6,10]. Given the diverse findings, we analyzed young consecutive MM patients at our CCCF. Notably, we did not find an association between age and specific disease characteristics, laboratory parameters at initial diagnosis, univariate risks, patients who died of PD, or comparative analyses (with 11 prior publications on younger MM cohorts; Table 2). In our cohort, the median PFS was 57 months, and the median OS was not reached. The 5- and 10-year OS rates were 83% and 72%, respectively. Despite these outcomes, the age-adjusted life expectancy for our patients, with a median age of 47 years at MM diagnosis, remained significantly reduced, indicating the need for a better prognosis.

Of note, previous studies had reported that low-risk ISS stages I and II positively impact PFS and OS in younger MM patients [2,3,7,10], while the presence of bone lesions, renal failure, or anemia negatively affects OS [2,8,22]. Some have also reported an elevated frequency of LCs only in younger MM patients [2,5,6,9,19]. Consistent with these findings, we observed that the majority of our patients were at early ISS stages (ISS I + II vs. III in 74% vs. 26%, respectively), with high frequencies of bone lesions (84%) and renal impairment (28%), although anemia was less common (38%). Notably, we are the first to report the R-ISS in younger MM patients, with most (78%) classified as R-ISS stage II/III. LC-only MM was observed in 33% of our patients, and unfavorable cytogenetics (t(4;14), t(14;16), t(14;20), del17p, hypoploidy, c-myc, or chromosome 1 aberrations) was present in 44%, while according to the International Myeloma Working Group (IMWG) criteria (del17p, t(14;16), t(4;14)), it was present in only 7%. The relatively high proportion of patients with unfavorable cytogenetics contributed to the higher R-ISS scores in our cohort. This suggests that the ISS staging system may not be fully reliable in this context, as it does not account for cytogenetics and that the R-ISS appears to provide a significantly more informative prognosis in this cohort. Notably, in our own prior analysis of MM patients across all age groups, we observed a lower incidence of unfavorable cytogenetics (32% as defined by our UKF high-risk criteria) compared to the 40% observed in our current study [15]. Additionally, a previous report examining an unselected cohort of MM patients revealed R-ISS distribution values (18%/64%/18%) [23] that closely mirror those found in our younger cohort here (22%/60%/18%). These findings suggest that, while the ISS may indicate a favorable prognosis for younger patients, the presence of high-risk cytogenetics may be a contributing factor to their more unfavorable prognosis.

As LC-only MM together with HRCG have been described as risk factors [2,5], we conducted bivariate analyses for both parameters and found HRs > 1 for PFS, but not for OS (Supplementary Table S2A). The frequency and role of HRCG in young patients have been inconclusively reported, with varying proportions reported across studies. In the studies by Pal et al. [18] and Jurczyszyn et al. [6], the proportions of patients ≤ 40 years with HRCG were reported to be as high as >30%. Conversely, in other studies, this proportion was much lower (10–21%; Table 2). Interestingly, in the aforementioned studies [6,18], HRCG was not identified as a univariate risk for worse prognosis, and younger patients exhibited better OS. However, in two other studies, where the proportion of HRCG was lower (14% and 18%), HRCG negatively impacted OS [2,5]. In another study, the frequency of t(11;14), which is generally considered standard risk, was found to be increased, with a worse outcome for PFS [19]. This observation was not verified by our data. Overall, further refined genetic analyses are warranted to better comprehend their role in determining outcomes in young MM patients.

Improved PFS and OS after tandem PBSCT, especially for HR patients, have been consistently reported [24,25,26,27,28]. However, allo-SCT is performed less frequently today due to its associated toxicity, including transplant-related mortality and adverse events, such as graft-versus-host disease [29,30,31,32], as well as due to other immunotherapies being currently available. Nevertheless, allo-SCT is still considered a potentially valid treatment for carefully selected subgroups of HR young and fit MM patients [16,33]. In our cohort, 51% of patients underwent at least two PBSCTs, some as tandem PBSCTs, and 31% including allo-SCT [16,34]. Due to the benefit of tandem PBSCTs, and allo-SCTs being extensively studied in MM (and because our subgroups receiving one or more PBSCTs differed in terms of individual time courses), we opted not to conduct further analyses.

The outcomes of younger MM patients can vary dramatically, as illustrated in Table 2: In two studies, specifically analyzing patients < 40 years treated during the same period, median PFS and OS were reported as 41 vs. 16 months and 175 vs. 61 months, respectively [2,9]. A likely reason for this variation seems to be the application of different therapy regimens, as shown by a recent study published during our manuscript preparation [35].

The strengths of our study were a consecutive and well-documented cohort of 68 young MM patients, comprehensive risk factor assessment, long-term follow-up, and thorough literature review. The limitations of our study were its retrospective nature and the fact that it was conducted at a single center. We did not make a comparison with a matched cohort of elderly patients, in line with previous studies (Table 2). It is important to note that defining an elderly comparative cohort would present significant challenges. A substantial proportion of our elderly patients are not candidates for ASCT, whereas all of our younger, fitter patients received HD therapy plus ASCT here. As a result, comparisons between these cohorts are inherently prone to errors and numerous imbalances. Furthermore, while our university center treats hundreds of myeloma patients annually, the number of younger patients remains relatively smaller, as evidenced in our report. Therefore, statistical analyses conducted at a single center lack the necessary statistical power for complex models incorporating adjustments for a considerable number of relevant prognostic factors. This is particularly true regarding events such as mortality, to adequately address the open questions posed in such studies. To address these limitations, we plan to extend our data through collaborative efforts, such as those within the German-speaking Myeloma Multicenter Group (GMMG)/Deutsche Studiengruppe Multiples Myelom (DSMM) and Medical Research Council (MRC).

Of note, during the preparation of our manuscript, Tanguay et al. and Steinbach et al. reported a similar review of manifestations and outcomes of younger MM patients [36,37]. In Tanguay’s report, the group concluded, similar to our study, that young MM patients tend to have a higher proportion of LC-only subtypes, lower ISS stages, and significant heterogeneity in cytogenetic abnormalities [36]. Steinbach et al. on the other hand conclude that young MM is usually associated with a better prognosis [37]. However, unlike both Tanguay et al. and Steinbach et al., we provided primary patient data including comprehensive cytogenetic analyses [36,37]. Additionally, none of the previous studies reported the R-ISS stage, which, in contrast to the ISS stage, appears to be increased in these patients.

## 5. Conclusions

In summary, MM in young patients remains unclear with no specific therapeutic interventions available that differ from the treatment of older MM patients, and no clear risk factors have been identified. It is therefore important that young patients are included in clinical trials, receiving innovative agent combinations, such as quadruplets and immunotherapy combinations. The question of whether younger MM patients benefit from very early and intensified therapeutic interventions remains unanswered. In the future, it is imperative that trials delve deeper into age-specific risks and refine therapies to better address the unique needs of young MM patients. Furthermore, cytogenetics and molecular testing in younger patients will be increasingly important in the future for a better understanding of their specific disease biology. These approaches are essential for advancing outcomes and enhancing the quality of life for this patient population. This crucial inquiry underscores the need for further research and clinical trials to elucidate the potential advantages of such approaches in this demographic.

## Figures and Tables

**Table 1:** Patient characteristics.

	n (%)	Median (Range)
**Age**		
29–50 years	68 (100)	47 (29–50)
29–40 years	11 (15)	39 (29–40)
**Gender**		
male–female (%)	41 (61):27 (39)	
**MM subtype**		
IgG/IgA/IgM/LC only/asecretory	33 (48)/11 (17)/0/23 (33)/1 (2)	
κ/λ/asecretory	45 (65)/22 (33)/1 (2)	
**ISS** I/II/III	29 (43)/21 (31)/18 (26)	
**R-ISS** I/II/III	15 (22)/41 (60)/12 (18)	
**Durie and Salmon** I/II/III	7 (10)/10 (15)/51 (75)	
A:B	53 (78)/15 (22)	
**Bone marrow plasma cell infiltration** (%)		50 (5–90)
**Cytogenetics UKF** * **/IMWG** ** (%)		
favorable	29 (43)/54 (80)	
unfavorable	30 (44)/5 (7)	
no aberrations	1 (1)/1 (1)	
missing	8 (12)/8 (12)	
**Number of CRAB criteria** ***		
C/R/A/B/none	13 (19)/19 (28)/26 (38)/57 (84)/5 (7)	
1/2/3/4/0	35 (51)/12 (18)/8 (12)/8 (12)/5 (7)	
**KPS (%)**		
100/90/80/≤70	8 (12)/20 (29)/23 (34)/17 (25)	80 (40–100)
**R-MCI**		
0–3 = fit/4–6 = intermediate fit/7–9 = frail	30 (44)/38 (56)/0 (0)	4 (0–6)
**Induction therapy**	52 (76)/8 (12)/5 (8)/3 (4)	
VCD/RAD/VRD/others		
**Transplantation**	32 (47)/15 (22)/21 (31)	
ASCT/tandem ASCT/ASCT+allo-SCT		
**Maintenance**	38 (56)/30 (44)	
yes (lenalidomide/bortezomib/carfilzomib)/no		
**Response to therapy**		
remission after induction	6 (9)/24 (35)/28 (41)/10 (15)	
CR/vgPR/PR/SD		
best remission during therapy	27 (40)/34 (50)/7 (10)/0	
CR/vgPR/PR/SD	32 (47)	
at least once progression (PD)		
**Outcome**	13 (19)	
PFS (months)		57 (2–124)
OS (months)		n.r. (10 n.r.)
death		

Abbreviations and definitions: n = number; Ig = immunoglobulin; LCs = light chains; ISS = International Staging System; R-ISS = Revised International Staging System; UKF = University of Freiburg; IMWG = International Myeloma Working Group; CRAB = calcium, renal insufficiency, anemia, bone lesions; KPS = Karnofsky Performance Status; R-MCI = Revised Myeloma Comorbidity Index; VCD = bortezomib, cyclophosphamide, dexamethasone; RAD = lenalidomide, adriamycin, dexamethasone; VRD = bortezomib, lenalidomide, dexamethasone; ASCT = autologous stem cell transplantation; allo-SCT = allogeneic stem cell transplantation. Response according to International Myeloma Working Group: CR = complete remission; vgPR = very good partial remission; PR = partial remission; SD = stable disease; PD = progressive disease; PFS = progression-free survival; OS = overall survival; n.r. = not reached. * Unfavorable UKF: t(4;14), t(14;16), t(14;20), del17p, hypoploidy, c-myc, chromosome 1 aberration. ** Unfavorable IMWG: del17p, t(14;16), t(4;14). *** more than 1 CRAB criteria possible, therefore in sum >100.

## Data Availability

The data underlying this article are available in the article and in its online Supplementary Materials. Further data supporting the findings of this study are available from the corresponding author upon reasonable request.

## Supplementary Materials

The following supporting information can be downloaded at https://www.mdpi.com/article/10.3390/cancers16234090/s1, Table S1A: Laboratory findings of young (≤50 years) MM patients at initial diagnosis; Table S1B: Peripheral blood reconstitution of leukocyte subsets in young (≤50 years) MM patients after ASCT; Table S2A: Cox regression, univariate and bivariate, in 68 patients; Table S2B: Univariate analysis of various risk parameters on PFS (5- and 10-year PFS estimates); Table S2C: Univariate analysis of various risk parameters on OS (5- and 10-year OS estimates); Table S3: Characteristics of patients who died of disease (MM) progression.
