# Supplementary material for: Clinical Characteristics, Cytogenetic Risks, and Prognoses of Young Multiple Myeloma Patients in the Era of Novel Therapies
Source: Cancers (Basel). 2024 Dec 6;16(23):4090. doi: 10.3390/cancers16234090 (PMC11640651; doi:10.3390/cancers16234090)
Supplement: Supplementary file 1 [file cancers-16-04090-s001.zip › cancers-3296646-supplementary.pdf]

Supplementary tables

Supplementary Table S1A. Laboratory findings of young (≤50 year) MM patients at initial diagnosis.

Laboratory	Median (range)	Normal range
Hemoglobin (g/dL)	11.6 (4.8-16.4)	m: 12-18; f: 11.6-15.5
Calcium (mmol/L)	2.4 (2-4.6)	2.15-2.55
Serum creatinine (mg/L)	1.0 (0.5-6)	0.67-1.17
eGFR (mL/min/1.73 m^2)	81 (11-174)	<30 - >120
ß2-microglobulin (mg/L)	3.5 (1-31.4)	0.8-2.2
Albumin (g/dL)	4.2 (2-30)	3.4-4.8
LDH (U/L)	201 (89-535)	135-225
Serum free LCs, kappa (k) or lambda (λ) (mg/L)	323 (2.3-48.700)	K: 3.3-19.4; λ: 5.7-26.3*

Supplementary Table S1B. Peripheral blood reconstitution of leukocyte subsets in young (≤50 year) MM patients after ASCT.

Immune cytology (FACS) assessment SCT	Median/µL (range)	Normal range/µL
Total leukocytes (/µL)	5,350 (4,890-5,210)	5,027-10,278
Total lymphocytes (/µL)	1,061 (45-3,336)	1,102-2,370
B-cells (/µL) (CD19+)	123 (10-749)	74-394
T-cells (/µL) (CD3+)	677 (83-2,695)	650-1864
T-helper cells (/µL) (CD3+CD4+)	286 (28-1,451)	424-1137
Cytotoxic T-cells (/µL) (CD3+CD8+)	295 (53-1,564)	199-746
T4:T8 ratio	0.7 (0.1-2.1)	1.0-3.3
Activated T-lymphocytes (/µL) (CD3+HLA-DR+)	62 (6-534)	23-296
NK cells (/µL) (CD3-CD16/56+)	139 (6-496)	150-600
NK T cells (/µL) (CD3+CD16/56+)	7 (1-80)	15-206

Abbreviations:
M: male, f: female;
N: number; eGFR: estimated glomerular filtration rate; LDH: lactate dehydrogenase; LC: light chains; FACS: fluorescence activated cell sorting; SCT: stem cell transplant; NK: natural killer; NKT: natural killer T
*reference range as defined by Binding site

Supplementary Table S2A. Cox regression, univariate and bivariate, in 68 patients.

			PFS			OS		
	**Risk factor**	**Risk group differentiation**	**HR**	**95% CI**	**p value**	**HR**	**95% CI**	**p value**
Univariate	LC-only MM	LC-only MM vs. others	1.273	[0.628, 2.582]	0.503	0.890	[0.274, 2.893]	0.846
	ISS	Stage II vs. Stage I	1.202	[0.532, 2.715]	0.658	2.062	[0.627, 6.782]	0.233
	ISS	Stage III vs. Stage I	1.205	[0.534, 2.716]	0.653	0.659	[0.128, 3.401]	0.618
	Cytogenetics	HR cytogenetics vs. standard risk	1.955	[0.667, 5.731]	0.221	0.710	[0.091, 5.517]	0.743
	CRAB criteria	3-4 criteria vs. < 3 criteria	0.942	[0.409, 2.172]	0.889	0.672	[0.149, 3.040]	0.606
	Anemia	Anemia vs. no anemia	1.004	[0.496, 2.033]	0.991	0.802	[0.247, 2.607]	0.713
	Bone lesions	Bone lesions vs. not existent	1.484	[0.571, 3.853]	0.418	1.289	[0.285, 5.837]	0.741
	Hypercalcemia	Hypercalcemia vs. no hypercalc..	1.319	[0.573, 3.041]	0.515	0.851	[0.188, 3.853]	0.833
	Renal impairment	Renal impairment vs. normal	0.743	[0.322, 1.712]	0.485	0.268	[0.035, 2.065]	0.206
	KPS	≤ 70% vs. >70%	1.160	[0.541, 2.489]	0.702	1.021	[0.279, 3.730]	0.975
	R-MCI	Intermediate fit vs. fit	1.381	[0.690, 2.765]	0.361	0.947	[0.317, 2.826]	0.922
	Induction: VCD	others vs. VCD	0.867	[0.391, 1.922]	0.725	0.820	[0.224, 3.001]	0.764
Bivariate	LC-only MM	LC-only vs. others	1.195	[0.569, 2.511]	0.637	0.658	[0.170, 2.545]	0.544
	HR cytogenetics	HR cytogenetics vs. standard risk	1.904	[0.645, 5.623]	0.243	0.837	[0.101, 6.935]	0.869

Abbreviations.:
PFS = progression free survival; OS = overall survival; HR = hazard ratio; CI = confidence interval; LC = light chain; MM = multiple myeloma; ISS = International Staging System; HR cytogenetics = high risk cytogenetics; CRAB = calcium, renal insufficiency, anemia, bone lesions; KPS = Karnofsky Performance Status; R-MCI = Revised Myeloma Comorbidity Index; VCD = bortezomib, cyclophosphamide, dexamethasone

Supplementary Table S2B. Univariate analysis of various risk parameters on PFS (5- and 10-year PFS estimates).

	5-year Survival Distribution Function Estimate (%) [95% CI]	10-year Survival Distribution Function Estimate (%) [95% CI]
**PFS after 1st ASCT**	49.9 [36.1, 62.3]	36.1 [22.3, 50.1]
**LC only**		
**Yes**	42.3 [18.7, 64.3]	n.r.
**No**	52.6 [35.7, 67.0]	40.8 [23.8, 57.1]
**ISS**		
**I**	47.8 [27.8, 65.3]	n.r.
**II**	49.7 [24.7, 70.4]	41.4 [17.5, 64.0]
**III**	56.6 [27.3, 77.9]	n.r.
**R-ISS**		
**I**	36.3 [11.7, 62.0]	n.r.
**II**	55.1 [37.0, 70.0]	46.5 [28.2, 63.0]
**III**	51.9 [19.8, 76.7]	n.r.
**D&S stage 3**		
**Yes**	39.2 [24.1, 54.0]	n.r.
**No**	80.1 [49.9, 93.2]	56.1 [21.2, 80.6]
**CG IMWG***		
**SR**	48.1 [32.2, 62.4]	30.6 [15.9, 46.7]
**HR**	n.r.	n.r.
**At least 1 CRAB criterium**		
**Yes**	48.7 [34.2, 61.7]	36.2 [21.7, 50.8]
**No**	60.0 [12.6, 88.2]	30.0 [1.2, 71.9]
**3-4 CRAB criteria**		
**Yes**	55.2 [22.1, 79.2]	n.r.
**No**	48.6 [33.4, 62.2]	38.5 [23.3, 53.5]
**Hypercalcemia****		
**Yes**	54.4 [21.9, 78.3]	n.r.
**No**	49.5 [34.2, 63.0]	39.2 [23.8, 54.2]
**Renal insufficiency*****		
**Yes**	63.8 [28.7, 85.1]	n.r.
**No**	45.4 [30.5, 59.2]	39.1 [24.4, 53.6]
**Anemia (<10 g/dL)**		
**Yes**	56.9 [32.4, 75.5]	n.r.
**No**	46.6 [30.0, 61.7]	38.0 [21.4, 54.5]
**Bone lesions**		
**Yes**	45.4 [30.4, 59.2]	35.2 [20.4, 50.3]
**No**	70.7 [33.7, 89.5]	42.4 [10.8, 71.9]
**KPS ≤70%**		
**Yes**	56.6 [27.3, 77.9]	n.r.
**No**	47.9 [32.1, 62.0]	39.8 [23.7, 55.4]
**R-MCI**		
**Fit**	54.9 [33.8, 71.7]	48.8 [27.4, 67.1]
**Intermediate fit**	46.6 [28.4, 63.0]	25.4 [9.6, 45.0]
**VCD induction therapy**		
**Yes**	53.2 [37.1, 66.9]	32.3 [16.2, 49.7]
**No**	41.7 [16.1, 65.7]	41.7 [16.1, 65.7]

Abbreviations:
95% CI: 95% confidence interval; PFS = Progression free survival; ASCT = autologous stem cell transplantation; LC = light chains; n.r. = not reached; ISS = International Staging System; R-ISS = Revised International Staging System; D&S = Durie&Salmon Staging System; CG = cytogenetics; IMWG = International Myeloma Working Group; SR = Standard risk; HR = High risk; KPS = Karnofsky Performance Status; R-MCI = Revised Myeloma Comorbidity Index; VCD = bortezomib, cyclophosphamide, dexamethasone
*Unfavorable cytogenetics via IMWG: del17p, t(14:16), t(4:14)
**Hypercalcaemia* (calcium >2.75 mmol/L)
***Renal insufficiency** (creatinine >2mg/dL)

Supplementary Table S2C. Univariate analysis of various risk parameters on OS (5- and 10-year OS estimates).

	5-year Survival Distribution Function Estimate (%) [95% CI]	10-yearSurvival Distribution Function Estimate (%) [95% CI]
**OS after 1st ASCT**	83.1 [70.8, 90.6]	72.1 [54.1, 83.9]
**LC only**		
**Yes**	88.7 [61.4, 97.1]	67.7 [30.8, 87.9]
**No**	80.6 [64.9, 89.8]	74.4 [54.0, 86.8]
**ISS**		
**I**	85.5 [65.6, 94.3]	n.r.
**II**	71.4 [44.2, 87.1]	59.5 [28.0, 81.0]
**III**	92.3 [56.6, 98.9]	83.9 [49.4, 95.7]
**R-ISS**		
**I**	76.6 [43.3, 91.9]	n.r.
**II**	83.8 [67.3, 92.4]	68.8 [42.1, 85.0]
**III**	90.0 [47.3, 98.5]	78.8 [38.1, 94.3]
**D&S stage 3**		
**Yes**	80.0 [65.0, 89.1]	65.0 [43.0, 80.2]
**No**	92.9 [59.1, 99.0]	92.9 [59.1, 99.0]
**CG IMWG***		
**SR**	80.5 [65.6, 89.4]	67.9 [45.3, 82.8]
**HR***	100 [100, 100]	n.r.
**At least 1 CRAB criterium**		
**Yes**	83.5 [70.6, 91.1]	73.2 [53.3, 85.7]
**No**	80.0 [20.4, 96.9]	60.0 [12.6, 88.2]
**3-4 CRAB criteria**		
**Yes**	82.5 [45.1, 95.5]	82.5 [45.1, 95.5]
**No**	83.2 [69.1, 91.2]	69.1 [47.7, 83.1]
**Hypercalcemia****		
**Yes**	82.5 [46.1, 95.3]	82.5 [46.1, 95.3]
**No**	83.5 [69.7, 91.4]	69.4 [47.9, 83.4]
**Renal insufficiency*****		
**Yes**	90.0 [47.3, 98.5]	90.0 [47.3, 98.5]
**No**	80.7 [66.1, 89.5]	66.9 [46.1, 81.2]
**Anemia (< 10 g/dL)**		
**Yes**	80.9 [56.2, 92.5]	80.9 [56.2, 92.5]
**No**	84.3 [68.2, 92.6]	67.2 [42.5, 83.1]
**Bone lesions**		
**Yes**	81.9 [67.9, 90.2]	70.1 [48.3, 84.0]
**No**	90.0 [47.3, 98.5]	78.8 [38.1, 94.3]
**KPS ≤70%**		
**Yes**	77.4 [44.9, 92.1]	77.4 [44.9, 92.1]
**No**	84.9 [70.8, 92.5]	71.6 [50.9, 84.7]
**R-MCI**		
**Fit**	81.3 [60.4, 91.9]	73.9 [48.7, 88.1]
**Intermediate fit**	84.6 [66.8, 93.3]	70.6 [42.4, 86.9]
**VCD induction therapy**		
**Yes**	79.9 [64.8, 89.1]	76.7 [60.6, 86.9]
**No**	92.9 [59.1, 99.0]	65.0 [22.8, 88.2]

Abbreviations:
95% CI: 95% confidence interval; PFS = Progression free survival; ASCT = autologous stem cell transplantation; LC = light chains; n.r. = not reached; ISS = International Staging System; R-ISS = Revised International Staging System; D&S = Durie&Salmon Staging System; CG = cytogenetics; IMWG = International Myeloma Working Group; SR = Standard risk; HR = High risk; KPS = Karnofsky Performance Status; R-MCI = Revised Myeloma Comorbidity Index; VCD = bortezomib, cyclophosphamide, dexamethasone

*Unfavorable cytogenetics via IMWG: del17p, t(14:16), t(4:14)

**Hypercalcaemia* (calcium >2.75 mmol/L)

***Renal insufficiency** (creatinine >2mg/dL)

Supplementary Table S3. Characteristics of patients who died of disease (MM) progression.

n = 12	Age	Sex	R-MCI	Ig type	ISS	CG	Therapy	Best remission	PD	PFS (mo)	OS (mo)
1	43	m	4	IgG kappa	II	HR	ASCT/alloSCT	vgPR	yes	16	25
2	43	m	4	LC kappa	I	HR	ASCT/alloSCT	vgPR	yes	44	94
3	42	m	6	IgG kappa	II	HR	ASCT/alloSCT	vgPR	yes	12	44
4	46	m	1	IgG kappa	II	SR	ASCT	PR	yes	18	654
5	49	f	3	LC kappa	I	NA	tandem ASCT	CR	yes	33	49
6	46	f	5	IgA lambda	II	HR	ASCT/alloSCT	vgPR	yes	9	27
7	47	m	2	IgA kappa	I	HR	ASCT	PR	yes	8	20
8	50	f	3	LC kappa	I	SR	ASCT/alloSCT	vgPR	yes	5	33
9	50	m	5	LC kappa	III	HR	tandem ASCT	vgPR	yes	27	634
10	49	f	5	IgG kappa	III	HR	ASCT	PR	yes	43	49
11	50	m	2	IgG lambda	II	SR	ASCT/alloSCT	vgPR	yes	2	10
12	48	m	2	IgG-kappa	II	SR	Tandem ASCT	vgPR	yes	52	88
Σ	Median 47	m:8 f:4	Median:4<br><br>Fit:6<br>Interm.-fit:6	IgG:6<br>IgA:2 LC only:4<br>K:λ:10:2	I:4<br>II:6<br>III:3	HR:1<br>SR:10<br>NA:1	ASCT:3<br>Tandem ASCT:3<br>ASCT/allo-SCT:6	CR:1<br>vgPR:8<br>PR:3	Yes:12	Median 39	Median 76

Abbreviations:
MM = multiple myeloma; n = number; R-MCI = Revised Myeloma Comorbidity Index; Ig = immunoglobuline; ISS = International Staging System; CG = cytogenetic; PD = progressive disease of MM; PFS = progression free survival; mo = months; OS = overall survival; m = male; HR = high risk; ASCT = autologous stem cell transplantation; allo-SCT = allogeneic stem cell transplantation; vgPR = very good partial remission; LC = light chains; SR = standard risk; PR = partial remission; f = female; NA = not available; CR = complete remission; Interm = intermediate
